# Aloin Induces Selective Cytotoxicity and Apoptotic Pathway Activation in Breast and Prostate Cancer Cells via Intrinsic and Extrinsic Mechanisms

**DOI:** 10.3390/ijms27125501

**Published:** 2026-06-18

**Authors:** Mohammadreza Dastouri, Buse Sanli

**Affiliations:** Department of Medical Biology, School of Medicine, Ankara Medipol University, Ankara 06570, Turkey; buse.sanli@std.ankaramedipol.edu.tr

**Keywords:** Aloin, Barbaloin, apoptosis, prostate cancer, breast cancer, cytotoxicity, intrinsic pathway, extrinsic pathway, PC-3, MCF-7

## Abstract

Breast and prostate cancers remain among the most prevalent epithelial malignancies worldwide, and conventional treatments often lack tumor selectivity. Aloin, an anthraquinone glycoside derived from Aloe vera, has demonstrated promising anticancer properties. This study investigated the differential cytotoxic and apoptotic effects of Aloin under in vitro conditions in MCF-7 (breast cancer) and PC-3 (prostate cancer) cell lines compared with normal prostate epithelial cells (PNT-A1). Cells were treated with Aloin (1000–1500 µg/mL); cytotoxicity was assessed by CCK-8 assay, apoptotic morphology by DIC microscopy, protein expression by immunofluorescence with quantitative CTCF analysis (BAX, Caspase-3, Caspase-8, Caspase-9), and gene expression by qRT-PCR (2^−ΔΔCt^ method). An integrated log_2_ fold change heatmap, pathway enrichment analysis across three independent databases (KEGG 2026, Reactome 2024, WikiPathways 2024), and STRING v12.0-based protein–protein interaction (PPI) network were constructed. Aloin exerted significant dose-dependent cytotoxicity in both cancer cell lines, while PNT-A1 viability exceeded 50% across all concentrations (Selectivity Index > 1.30 for MCF-7 at 48 h). Immunofluorescence and qRT-PCR confirmed significant upregulation of BAX (up to 6.14×), CASP8 (up to 15.51×), CASP9 (up to 9.27×), and CASP3 (3.03× in PC-3), indicating concurrent activation of intrinsic and extrinsic apoptotic pathways, while all genes remained unchanged in PNT-A1 cells. Pathway enrichment analysis confirmed that these genes are statistically central nodes in conserved apoptotic signaling networks (adj. *p* < 10^−9^). To the best of our knowledge, this is the first in vitro characterization of Aloin-induced pro-apoptotic activity in prostate cancer cells, establishing a mechanistic foundation for further investigation of this phytochemical in epithelial-derived cancer models.

## 1. Introduction

Cancer represents a heterogeneous group of diseases characterized by uncontrolled cell proliferation, invasion, and metastasis across various organs and tissues in the human body [1,2]. Globally, it remains one of the leading causes of mortality, accounting for approximately one in six deaths in 2020 [1]. According to Cancer Today 2022 statistics, breast and prostate cancers rank among the most prevalent malignancies worldwide. Prostate cancer is the second most common cancer in men, with 1,467,854 reported cases, while breast cancer is both the most frequently diagnosed and deadliest cancer among women, with 2,296,840 new cases [3]. Despite decades of advances in cancer therapy, current treatments often fail to achieve complete remission and are limited by toxicity, drug resistance, and relapse [4].

Conventional treatment strategies for prostate cancer—including androgen deprivation therapy, radical prostatectomy, and radiotherapy—are associated with substantial limitations. These include the development of castration resistance [5,6], treatment-related metastasis and recurrence [7], and persistently elevated prostate-specific antigen (PSA) levels [8]. Likewise, therapeutic approaches for breast cancer vary by stage and molecular subtype, encompassing chemotherapy, endocrine therapy, immunotherapy, bone-modifying agents, and surgical interventions such as lumpectomy or mastectomy [9]. However, the cytotoxicity of these treatments toward normal cells and the emergence of multidrug resistance underscore the need for alternative therapeutic modalities, particularly those of natural origin.

Natural compounds derived from medicinal plants have long been utilized in traditional medicine and remain an invaluable source of anticancer agents [10]. Remarkably, more than 60% of current anticancer drugs are derived from natural sources [11]. Among these bioactive molecules, Aloin—also known as Barbaloin—is an anthraquinone glycoside (C_21_H_22_O_9_) composed of two diastereoisomers, Aloin A and Aloin B, which are C-glycoside derivatives of aloe-emodin anthrone [12]. Beyond its well-known laxative [13], anti-inflammatory [14], antimicrobial [15,16], antioxidant [17], and antidiabetic [18] effects, Aloin has also been shown to exert potent anticancer and antitumor activities [19,20,21,22,23].

Aloin’s anticancer potential has been explored across multiple tumor models. For instance, Pan et al. (2013) demonstrated that Aloin inhibited proliferation, migration, and tube formation in colorectal cancer by suppressing VEGF receptor and STAT3 phosphorylation [24]. In gastric cancer, Aloin reduced proliferation and migration through inhibition of Akt/mTOR, STAT3, and NF-κB pathways [25]. Similarly, in non-small-cell lung cancer, Aloin decreased cell viability in a dose-dependent manner and induced apoptosis in A549 and H1299 cells [26]. In melanoma, Aloin was shown to suppress HMGB1 expression and potentially modulate the ERK pathway [27]. More recently, studies have indicated that Aloin interferes with the PI3K/AKT signaling axis and promotes apoptosis in gastric cancer [28]. Notably, Aloin has been shown to induce apoptosis and cell-cycle arrest in breast cancer cell lines (MCF-7, T47D, SKBR3) [20,29] and to promote autophagy in MCF-7 cells [30] and to alleviate doxorubicin-induced cardiotoxicity in experimental models [31].

Collectively, these findings support Aloin’s role as a promising multi-target natural compound with potent anti-proliferative and pro-apoptotic effects in diverse malignancies. However, to date, no studies have investigated the cytotoxic and apoptotic effects of Aloin on prostate cancer cells.

To fill this critical gap, the present study aimed to comprehensively evaluate Aloin’s cytotoxic and apoptotic effects in prostate cancer cells (PC-3) for the first time, using PNT-A1 normal prostate epithelial cells as the matched normal comparator. MCF-7 breast cancer cells were included as a well-characterized positive reference line, given that Aloin’s antiproliferative activity in this cell line has been previously reported in the literature [20,29,30], thereby providing an internal benchmark against which the novel prostate cancer findings could be contextualized. Both cell lines are of epithelial origin, consistent with the use of PNT-A1 normal prostate epithelial cells as the non-malignant comparator. The apoptotic mechanism was elucidated through immunofluorescence analysis of apoptosis-related proteins with quantitative CTCF measurements, and quantitative real-time PCR (qRT-PCR) gene expression profiling, alongside pathway enrichment analysis and protein–protein interaction network construction.

## 2. Results

### 2.1. Aloin-Induced Morphological Changes Observed by DIC Microscopy

DIC microscopy revealed clear morphological differences between Aloin-treated and untreated cells, as well as distinct responses in cancerous (PC-3, MCF-7) versus normal prostate epithelial (PNT-A1) cells. In untreated cancer cells, the monolayer structure remained intact, with cells displaying normal adherence, compact cytoplasmic appearance, and uniform nuclear morphology. Following Aloin treatment, cancer cells showed pronounced apoptotic alterations including cellular rounding, increased optical contrast, and loss of substrate adherence. Treated cancer cells also exhibited marked nuclear fragmentation and the appearance of numerous apoptotic bodies, indicating advanced apoptotic progression and clear disruption of monolayer organization.

In untreated PNT-A1 normal cells, morphology remained stable with intact epithelial structure and homogeneous nuclear appearance. After Aloin exposure, PNT-A1 cells demonstrated only mild morphological changes, with mostly preserved cell shape and adherence. Nuclear fragmentation was minimal and apoptotic body formation was limited, indicating that normal cells were substantially more resistant to Aloin-induced apoptotic damage compared with cancer cells.

**Figure 1:**
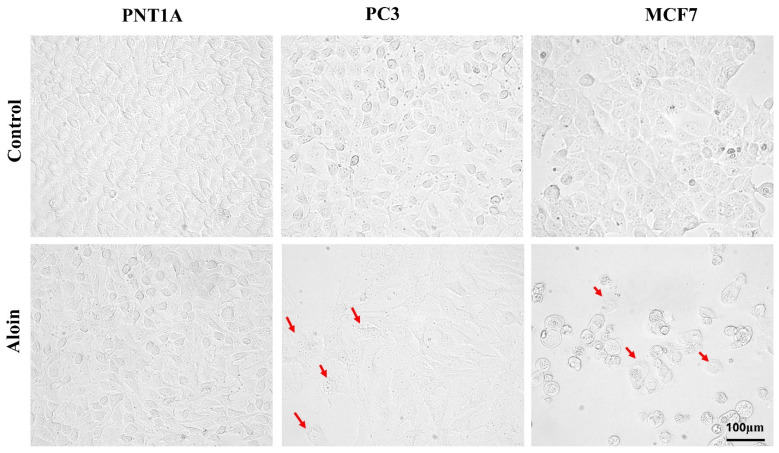
DIC microscopy images showing the morphological response of normal and cancer cells to Aloin treatment (1250 µg/mL, 48 h). PNT-A1 normal epithelial cells exhibited preserved morphology under both control and Aloin-treated conditions, maintaining typical epithelial appearance and intact monolayer structure. In contrast, untreated PC-3 and MCF-7 cancer cells displayed normal, confluent growth patterns, whereas Aloin-treated groups showed reduced cell density and clear apoptotic morphology. Apoptotic features—including cell rounding, loss of adherence, and formation of apoptotic bodies—are highlighted by red arrows in PC-3 and MCF-7 panels. Images were captured by DIC microscopy (magnification: 20×). Scale bar = 100 µm (applies to all panels).

Overall, DIC imaging showed that Aloin preferentially induced prominent apoptotic morphology in cancerous cells—characterized by cell enlargement, nuclear fragmentation, and apoptotic body formation—whereas normal epithelial cells exhibited markedly preserved structural integrity (Figure 1).

### 2.2. Cytotoxic Effects of Aloin Assessed by CCK-8 Assay

**Figure 2:**
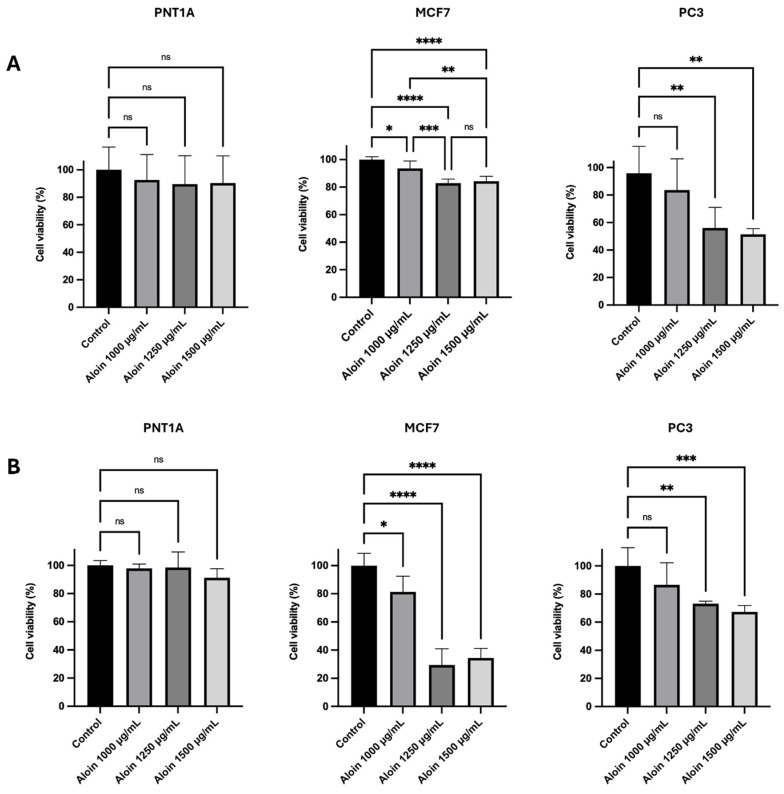
(**A**) Cell viability of PNT-A1, MCF-7, and PC-3 cells following 24 h exposure to increasing concentrations of Aloin (1000, 1250, and 1500 µg/mL). PNT-A1 cells showed no statistically significant changes across treatments, whereas MCF-7 and PC-3 cells exhibited significant reductions in viability at multiple concentrations (*p* < 0.05 to *p* < 0.0001). (**B**) Cell viability of PNT-A1, MCF-7, and PC-3 cells after 48 h Aloin treatment. PNT-A1 cells showed no significant alterations. MCF-7 cells displayed further reductions in viability compared with 24 h, with several concentrations reaching high statistical significance (*p* < 0.01 to *p* < 0.0001). PC-3 cells exhibited dose-dependent cytotoxicity at 48 h, although the cytotoxic response was attenuated compared to 24 h, consistent with the adaptive phenotype of this androgen-independent cell line. Data are presented as mean ± SD from three independent experiments. Statistical analysis was performed using one-way ANOVA with Tukey’s post hoc test. ns = not significant; * *p* < 0.05; ** *p* < 0.01; *** *p* < 0.001; **** *p* < 0.0001.

CCK-8 cell viability assays demonstrated that Aloin exerted dose-dependent cytotoxicity in both cancer cell lines tested. In PNT-A1 normal epithelial cells, viability remained stable across all concentrations at both 24 h and 48 h, with no statistically significant differences relative to untreated controls (Figure 2A,B). In contrast, MCF-7 cells exhibited significant reductions in viability beginning at lower Aloin concentrations, with further decreases observed at higher doses. This effect was evident at 24 h and became more pronounced after 48 h, where several concentrations reached strong statistical significance (*p* < 0.05 to *p* < 0.0001). PC-3 cells demonstrated significant loss of viability at 24 h across all tested concentrations (*p* < 0.01 to *p* < 0.001). Notably, at 48 h, PC-3 cells exhibited attenuated cytotoxic responses compared to 24 h, a pattern distinct from MCF-7 cells, which is discussed further in relation to PC-3 cell biology and Aloin stability characteristics.

### 2.3. IC50 and Selectivity Index (SI) Analysis

IC50 analysis revealed that Aloin did not reduce PNT-A1 cell viability to 50% at either 24 h or 48 h, indicating IC50 values above 1.5 mg/mL at both time points. In MCF-7 cells, the IC50 exceeded 1.5 mg/mL after 24 h but decreased to 1.15 mg/mL after 48 h, indicating enhanced sensitivity with prolonged exposure. In PC-3 cells, the IC50 was estimated at 1.49 mg/mL at 24 h, whereas at 48 h it remained above 1.5 mg/mL. SI analysis indicated modest selectivity only under specific conditions, with the most notable value observed in MCF-7 cells after 48 h exposure (SI > 1.30). SI values should be interpreted cautiously as minimum estimates; however, the observed SI > 1.30 for MCF-7 at 48 h suggests preferential cytotoxicity toward cancer cells under prolonged exposure conditions (Table 1).

### 2.4. Pathway Enrichment Analysis

**Figure 3:**
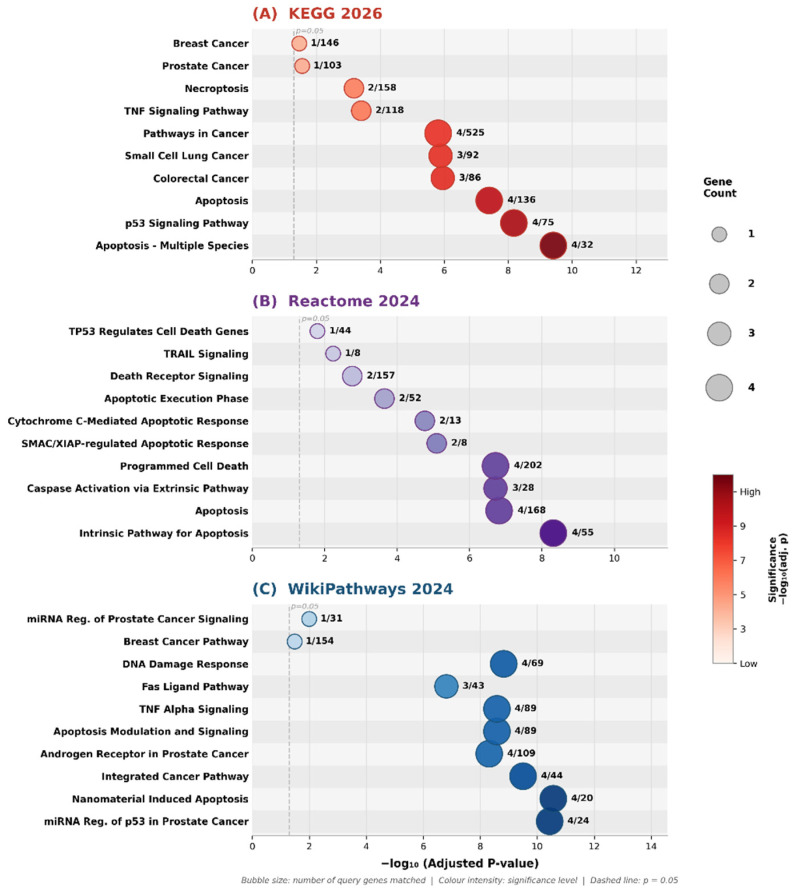
Pathway enrichment analysis of the four apoptosis-related genes targeted by Aloin (BAX, CASP3, CASP8, CASP9) across three independent databases: KEGG 2026, Reactome 2024, and WikiPathways 2024 Human. Analysis was performed using the Enrichr web platform. Each row represents a significantly enriched biological pathway (adjusted *p*-value < 0.05). Bubble size indicates the number of query genes matched to each pathway (gene overlap); color intensity reflects statistical significance expressed as −log_10_ (adjusted *p*-value), with darker colors representing greater significance. The dashed vertical line marks the *p* = 0.05 significance threshold. Pathways are ranked by statistical significance within each database panel. Adjusted *p*-values were calculated using Fisher’s exact test with Benjamini–Hochberg correction. Each database panel is displayed in a distinct color scheme: KEGG 2026 (red), Reactome 2024 (purple), and WikiPathways 2024 Human (blue).

To guide the selection of target genes for subsequent experimental analyses, pathway enrichment analysis was first performed using the Enrichr platform across three independent databases (KEGG 2026, Reactome 2024, WikiPathways 2024 Human) (Figure 3). This bioinformatic approach identified BAX, CASP3, CASP8, and CASP9 as statistically central nodes within conserved apoptotic signaling networks, providing a rational basis for their selection as experimental targets. In KEGG 2026, all four genes were significantly enriched in Apoptosis—Multiple Species (adj. *p* = 3.94 × 10^−10^) and p53 Signaling Pathway (adj. *p* = 6.65 × 10^−9^). Notably, both Prostate Cancer (adj. *p* = 0.028) and Breast Cancer (adj. *p* = 0.034) pathways were significantly enriched, providing independent bioinformatic support for the cancer type selection in this study. Reactome 2024 analysis confirmed concurrent activation of intrinsic and extrinsic apoptotic pathways, with Intrinsic Pathway for Apoptosis (adj. *p* = 4.91 × 10^−9^) and Caspase Activation via Extrinsic Apoptotic Signalling Pathway (adj. *p* = 1.94 × 10^−7^) among the most significant terms. WikiPathways 2024 analysis further revealed enrichment of miRNA Regulation of p53 Pathway in Prostate Cancer (adj. *p* = 3.71 × 10^−11^) and Androgen Receptor Network in Prostate Cancer (adj. *p* = 4.85 × 10^−9^), reinforcing the relevance of these genes to prostate cancer biology. Collectively, these findings confirm that BAX, CASP3, CASP8, and CASP9 are statistically central nodes in conserved apoptotic signaling networks across multiple independent databases.

### 2.5. Gene Expression Analysis by qRT-PCR

**Figure 4:**
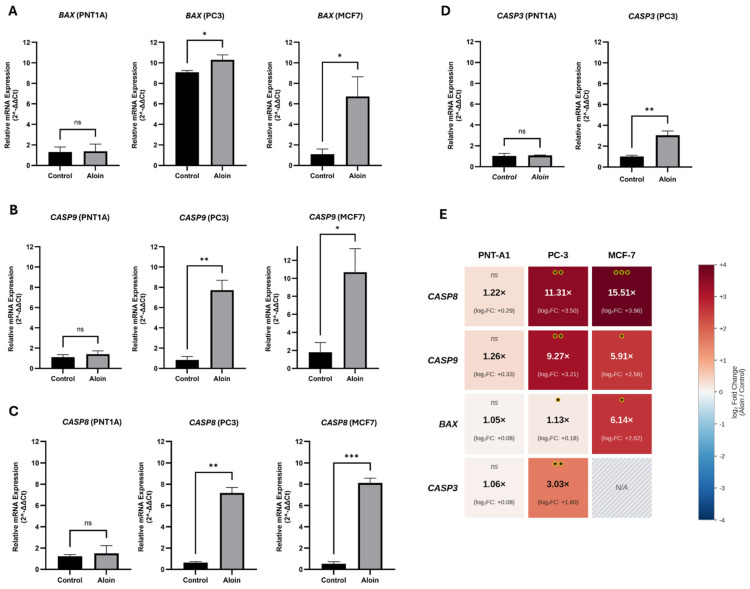
(**A**–**D**) Quantitative real-time PCR (qRT-PCR) analysis of apoptotic gene expression in PNT-A1 (normal prostate epithelial), PC-3 (prostate cancer), and MCF-7 (breast cancer) cells following 48 h treatment with 1250 µg/mL Aloin. Gene expression was normalized to GAPDH using the 2^−ΔΔCt^ method. (**A**) CASP9 mRNA was significantly upregulated in Aloin-treated PC-3 (*p* < 0.01) and MCF-7 (*p* < 0.05) cells, while PNT-A1 cells showed no significant change. (**B**) BAX mRNA levels were significantly elevated in PC-3 (*p* < 0.05) and MCF-7 (*p* < 0.05) cells, with no significant alteration in PNT-A1 cells. (**C**) CASP8 mRNA expression was significantly increased in PC-3 (*p* < 0.01) and MCF-7 (*p* < 0.001) cells, with no significant effect in PNT-A1 cells. (**D**) CASP3 mRNA was analyzed exclusively in PNT-A1 and PC-3 cells; CASP3 was significantly upregulated in PC-3 (*p* < 0.01), while PNT-A1 cells showed no significant change. CASP3 analysis was excluded from MCF-7 cells due to the known 47 bp deletion in exon 3 of the CASP3 gene, which abrogates functional Caspase-3 protein expression in this cell line. (**E**) Heatmap illustrating log_2_ fold change (Aloin-treated vs. vehicle control, 2^−ΔΔCt^ method) for all four apoptotic genes across PNT-A1, PC-3, and MCF-7 cell lines. Color intensity represents the magnitude of transcriptional response on a diverging Red–Blue scale: deep red indicates strong upregulation, white indicates no change, and blue indicates downregulation relative to control. Fold change (×) and log_2_FC values are annotated within each cell. N/A: CASP3 not assessed in MCF-7 (47 bp exon 3 deletion). All data are presented as mean ± SD from three independent experiments (n = 3). Statistical significance was determined by one-way ANOVA with Tukey’s post hoc test. * *p* < 0.05; ** *p* < 0.01; *** *p* < 0.001; ns: not significant.

To evaluate the effect of Aloin on apoptotic gene expression at the transcriptional level, qRT-PCR analysis was performed to assess the mRNA expression of CASP9, BAX, CASP8, and CASP3 in Aloin-treated and control cells (Figure 4A–D). The log_2_ fold change values across all genes and cell lines are further visualized in the heatmap (Figure 4E), providing an integrated overview of the differential transcriptional response to Aloin. In PNT-A1 normal epithelial cells, the expression of all analyzed genes remained unchanged following Aloin treatment, with no statistically significant differences between control and treated groups (*p* > 0.05, ns), confirming the absence of apoptotic gene induction in normal cells.

In PC-3 prostate cancer cells, Aloin treatment resulted in significant upregulation of all four apoptotic genes. CASP9 mRNA expression was significantly increased compared to control (*p* < 0.01), indicating activation of the intrinsic apoptotic pathway. BAX expression was similarly elevated in Aloin-treated PC-3 cells (*p* < 0.05), consistent with mitochondria-mediated apoptosis. Extrinsic pathway activation was evidenced by a significant increase in CASP8 mRNA levels (*p* < 0.01). Furthermore, CASP3 expression was significantly upregulated in PC-3 cells following Aloin treatment (*p* < 0.01), demonstrating downstream effector caspase activation.

In MCF-7 breast cancer cells, Aloin significantly induced CASP9 mRNA expression (*p* < 0.05), supporting intrinsic pathway activation. BAX mRNA levels were also significantly elevated (*p* < 0.05). CASP8 expression showed a highly significant increase (*p* < 0.001), indicating robust extrinsic pathway activation. CASP3 mRNA analysis was not performed in MCF-7 cells due to the well-characterized 47 bp deletion in exon 3 of the CASP3 gene in this cell line, which results in the absence of functional Caspase-3 protein expression. Collectively, these findings demonstrate that Aloin preferentially upregulates apoptotic gene expression at the mRNA level in cancer cells while PNT-A1 normal epithelial cells showed no statistically significant response under identical in vitro conditions.

### 2.6. Analysis of Apoptosis-Related Proteins by Immunofluorescence

**Figure 5:**
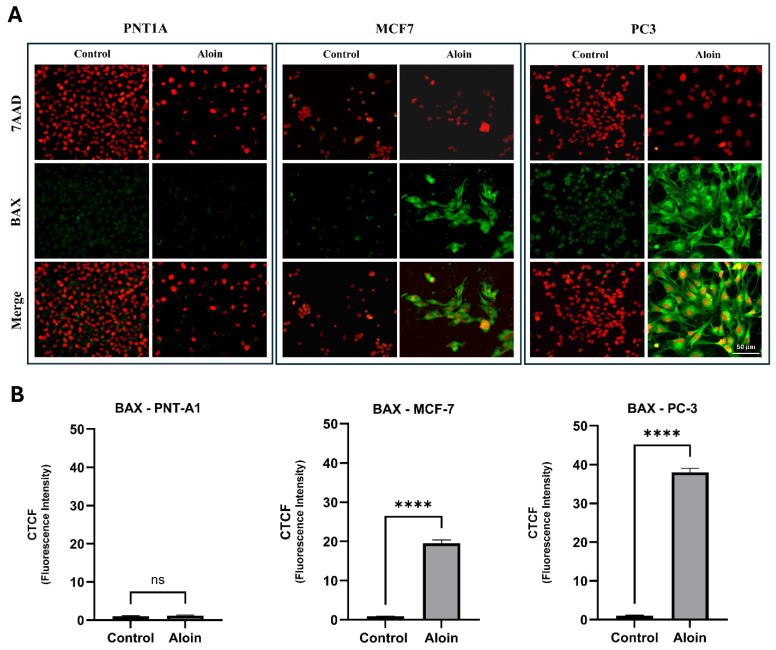
Immunofluorescence analysis of BAX expression in PNT-A1, MCF-7, and PC-3 cells following Aloin treatment (1250 µg/mL, 48 h). (**A**) Representative fluorescence images showing BAX protein (FITC, green) and nuclear staining (7-AAD, red) in control and Aloin-treated cells. PNT-A1 samples exhibited low BAX signal in both groups with preserved morphology. Aloin-treated MCF-7 and PC-3 cells showed marked increases in green fluorescence along with enlarged cell morphology and reduced monolayer density relative to controls. Images acquired at 40× magnification. Scale bar = 50 µm (applies to all panels). (**B**) Quantitative fluorescence intensity analysis by Corrected Total Cell Fluorescence (CTCF) calculated using ImageJ software. CTCF values are presented as mean ± SD (n = 10 cells per group). Statistical comparison between control and Aloin-treated groups was performed using unpaired Student’s *t*-test. ns = not significant; **** *p* < 0.0001.

Immunofluorescence staining demonstrated distinct, Aloin-dependent modulation of apoptotic protein expression across normal and cancer cell lines. In PNT-A1 cells, both control and treated groups retained a homogeneous red nuclear profile (7-AAD staining) with minimal green fluorescence (FITC), indicating that Aloin did not substantially alter apoptotic protein levels or cellular structure (Figure 5). In contrast, MCF-7 cells displayed a marked increase in green fluorescence following Aloin treatment, reflecting strong induction of apoptotic signaling. This was accompanied by reduced cell density and enlarged, irregular cell morphology, consistent with growth arrest and early apoptotic commitment. A similar but more pronounced pattern was observed in PC-3 cells, where Aloin exposure elicited intense and widespread green fluorescence, together with striking cellular enlargement and disrupted monolayer organization.

**Figure 6:**
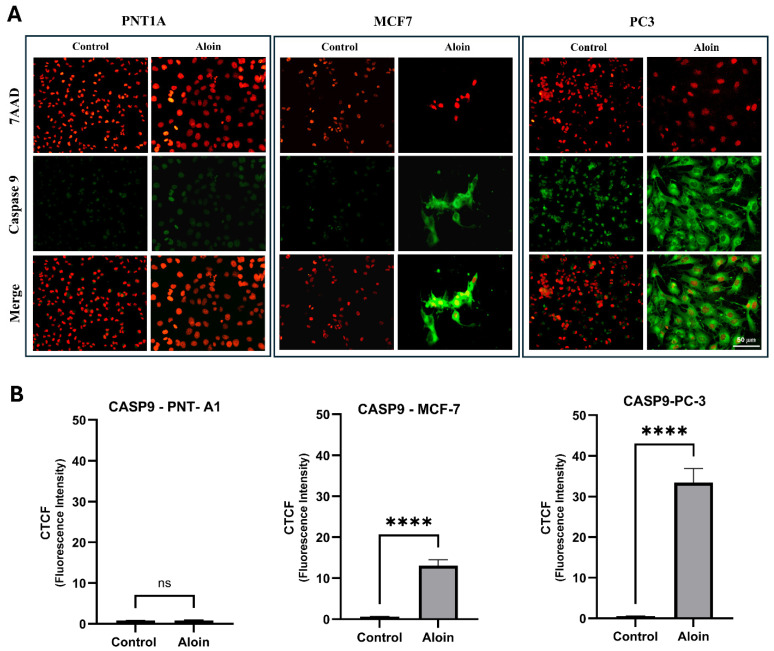
Immunofluorescence analysis of Caspase-9 expression in PNT-A1, MCF-7, and PC-3 cells following Aloin treatment (1250 µg/mL, 48 h). (**A**) Representative fluorescence images showing Caspase-9 protein (FITC, green) and nuclear labeling (7-AAD, red) in untreated and Aloin-treated cells. PNT-A1 samples maintained low Caspase-9 fluorescence in both groups. Aloin-treated MCF-7 cells exhibited strong, concentrated fluorescent regions, whereas PC-3 cells displayed diffuse and uniformly increased Caspase-9 signal across the monolayer. Cancer cells also showed enlarged morphology and reduced confluency after treatment. Images obtained at 40× magnification. Scale bar = 50 µm (applies to all panels). (**B**) Quantitative CTCF analysis using ImageJ software. CTCF values are presented as mean ± SD (n = 10 cells per group). Statistical comparison between control and Aloin-treated groups was performed using unpaired Student’s *t*-test. ns = not significant; **** *p* < 0.0001.

Extending the analysis to additional apoptotic regulators, intrinsic pathway markers BAX and Caspase-9 showed minimal changes in PNT-A1 but displayed substantial treatment-associated intensification in both MCF-7 and PC-3 cells (Figure 5 and Figure 6).

**Figure 7:**
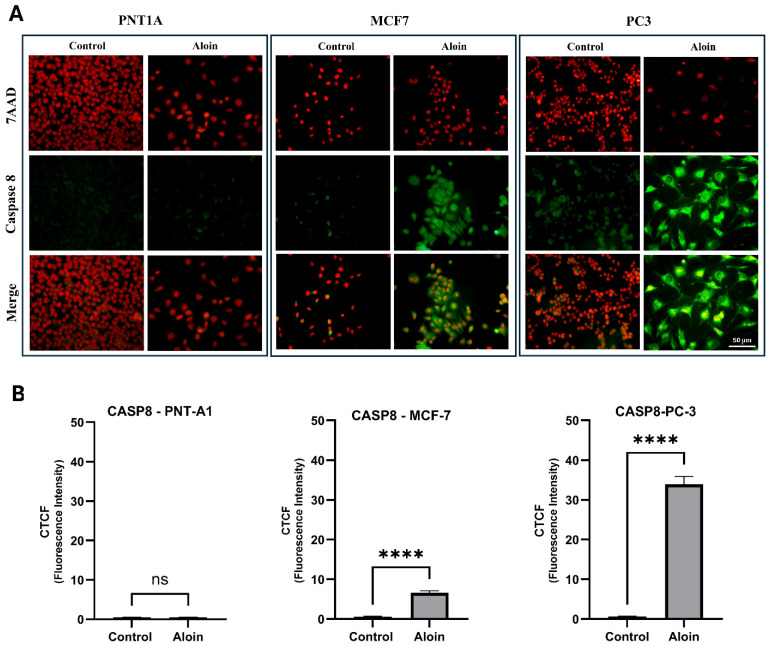
Immunofluorescence analysis of Caspase-8 expression in PNT-A1, MCF-7, and PC-3 cells following Aloin treatment (1250 µg/mL, 48 h). (**A**) Representative fluorescence images showing Caspase-8 protein (FITC, green) and 7-AAD nuclear counterstaining (red) in control and Aloin-treated groups. PNT-A1 cells displayed weak and unchanged Caspase-8 signal across conditions. Aloin-treated MCF-7 and PC-3 cells exhibited enhanced green fluorescence, with MCF-7 showing discrete intensified regions and PC-3 demonstrating more widespread cytoplasmic signal. Treated cancer cells also showed increased cell size and decreased cell density. Images captured at 40× magnification. Scale bar = 50 µm (applies to all panels). (**B**) Quantitative CTCF analysis using ImageJ software. CTCF values are presented as mean ± SD (n = 10 cells per group). Statistical comparison between control and Aloin-treated groups was performed using unpaired Student’s *t*-test. ns = not significant; **** *p* < 0.0001.

**Figure 8:**
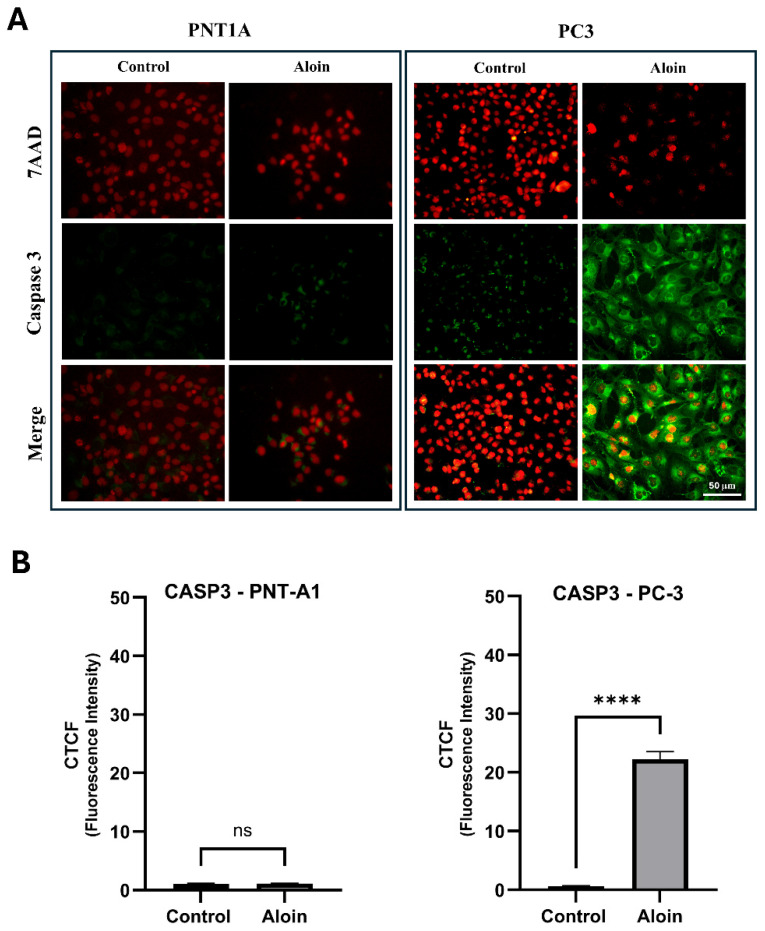
Immunofluorescence analysis of Caspase-3 expression in PNT-A1 and PC-3 cells following Aloin treatment (1250 µg/mL, 48 h). (**A**) Representative fluorescence images showing Caspase-3 protein (FITC, green) and nuclear staining (7-AAD, red) in control and Aloin-treated samples. PNT-A1 cells exhibited minimal Caspase-3 signal with no appreciable treatment-related change. PC-3 cells showed strong and widespread Caspase-3 staining throughout the treated monolayer, consistent with downstream effector caspase activation. Caspase-3 immunofluorescence was not performed in MCF-7 cells due to the known CASP3 gene deletion, which results in the absence of functional Caspase-3 protein. Images acquired at 40× magnification. Scale bar = 50 µm (applies to all panels). (**B**) Quantitative CTCF analysis using ImageJ software. CTCF values are presented as mean ± SD (n = 10 cells per group). Statistical comparison between control and Aloin-treated groups was performed using unpaired Student’s *t*-test. ns = not significant; **** *p* < 0.0001.

Likewise, examination of the extrinsic pathway marker Caspase-8 revealed a prominent rise in green fluorescence in Aloin-treated cancer cells while remaining largely unchanged in PNT-A1 (Figure 7). Finally, downstream Caspase-3 activation was assessed exclusively in PC-3 cells, given the well-characterized absence of functional Caspase-3 protein in MCF-7 cells due to a genomic deletion in the CASP3 gene. PC-3 cells exhibited a strong fluorescent increase in Caspase-3 signal following Aloin treatment, consistent with progression toward execution-phase apoptosis, whereas PNT-A1 cells showed negligible differences between groups (Figure 8).

Collectively, these findings show that Aloin triggers a broad apoptotic response in cancer cells through simultaneous activation of intrinsic and extrinsic apoptotic regulators, while normal epithelial cells remain largely unaffected at both the morphological and protein expression level.

**Figure 9:**
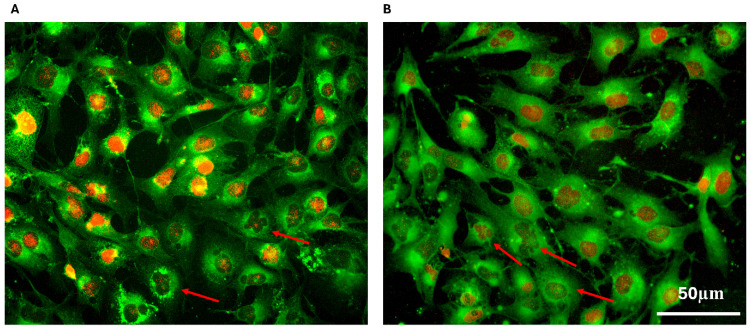
High-magnification immunofluorescence images demonstrating apoptotic morphology in Aloin-treated PC-3 prostate cancer cells (1250 µg/mL, 48 h). (**A**) Caspase-3 immunofluorescence (FITC, green) with 7-AAD nuclear counterstaining (red). (**B**) Caspase-9 immunofluorescence (FITC, green) with 7-AAD nuclear counterstaining (red). Both panels show characteristic apoptotic features including intense cytoplasmic caspase signal accumulation, nuclear DNA fragmentation evidenced by irregular and condensed 7-AAD staining, altered cell morphology with loss of normal cellular architecture, and markedly elevated protein expression relative to untreated controls. The co-localization of cytoplasmic caspase signals with fragmented nuclear staining provides morphological evidence consistent with caspase-mediated apoptotic cell death rather than necrosis, the latter being characterized by cellular swelling and membrane rupture, which are absent in these images. Images acquired at 40× magnification. Scale bar = 50 µm (applies to all panels). Red arrows indicate representative cells exhibiting apoptotic morphology.

To further substantiate the apoptotic nature of the observed cell death, high-magnification immunofluorescence images of Aloin-treated PC-3 cells are presented in Figure 9. These images demonstrate characteristic apoptotic morphological features—including intense cytoplasmic Caspase-3 and Caspase-9 signal accumulation, nuclear DNA fragmentation evidenced by irregular and condensed 7-AAD staining, and loss of normal cellular architecture—which are morphologically distinct from necrosis, the latter being characterized by cellular swelling and membrane rupture that are absent in these images.

### 2.7. Protein–Protein Interaction Network of Aloin-Targeted Apoptotic Genes

**Figure 10:**
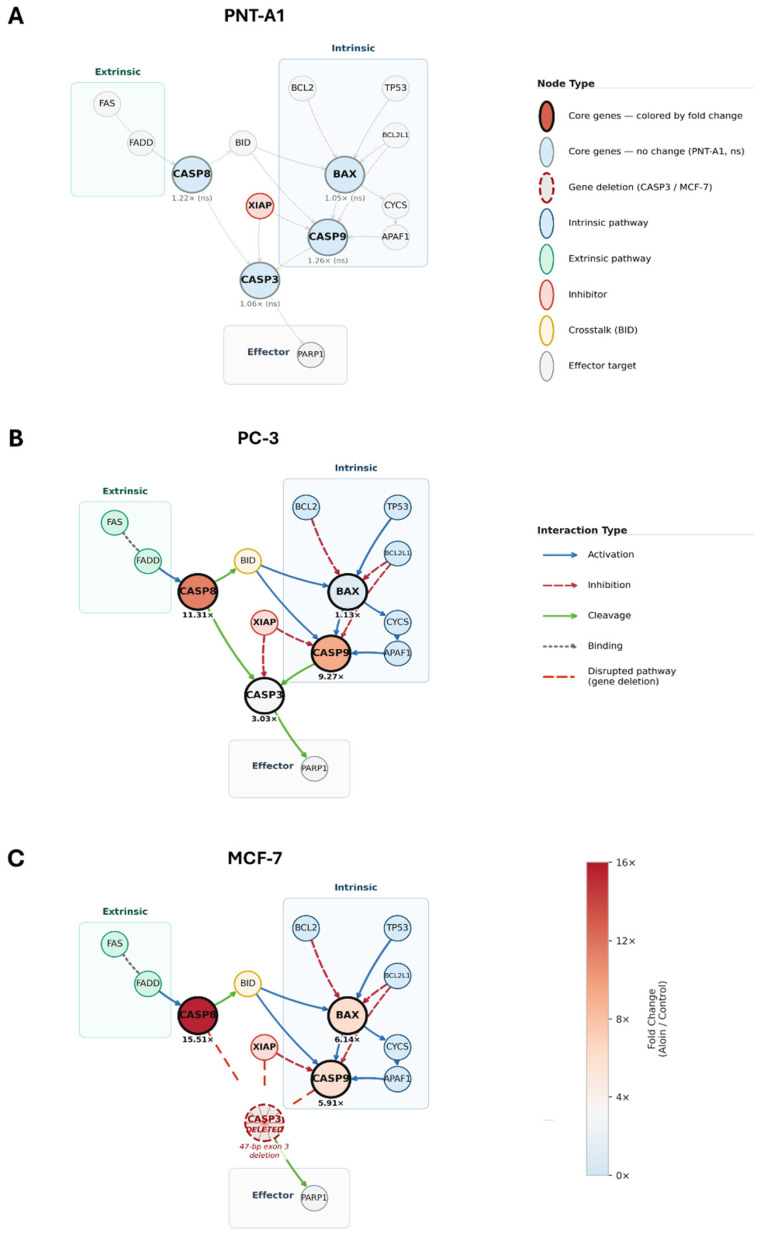
Protein–protein interaction (PPI) network of apoptosis-related genes following Aloin treatment (1250 µg/mL, 48 h), constructed using STRING v12.0 (Homo sapiens; combined confidence score ≥ 0.700) with first-degree interactors. (**A**) PNT-A1 normal prostate epithelial cells. (**B**) MCF-7 breast cancer cells. (**C**) PC-3 prostate cancer cells. Core gene nodes (CASP8, CASP9, BAX, CASP3) are colored according to fold change relative to vehicle control (qRT-PCR); color intensity from light blue (no change) to deep red (high upregulation). Grey-blue nodes in PNT-A1 (**A**) indicate no significant change (ns). Red dashed lines in MCF-7 (**B**) indicate disrupted downstream signaling due to the known 47 bp exon 3 deletion in the CASP3 gene. Arrow types indicate interaction category: blue solid = activation; red dashed = inhibition; green solid = proteolytic cleavage; grey dotted = binding. Edge thickness reflects STRING combined confidence score.

To position the experimentally validated genes within the broader apoptotic signaling network, a STRING v12.0-based PPI network analysis was performed. The network revealed that all four Aloin-targeted genes—CASP8, CASP9, BAX, and CASP3—occupy central nodes within the human apoptosis network, interconnected through both intrinsic and extrinsic pathway components (Figure 10). In PNT-A1 normal cells, all four core genes showed negligible fold change (1.05–1.26×), reflected by pale coloring across all network nodes. In contrast, PC-3 and MCF-7 cancer cells displayed pronounced upregulation of CASP8 (11.31× and 15.51×, respectively) and CASP9 (9.27× and 5.91×, respectively), visualized as intensely red core nodes, confirming robust activation of both apoptotic arms. The network further highlights the crosstalk node BID, which bridges the extrinsic (CASP8) and intrinsic (BAX–CYCS–APAF1–CASP9) pathways, as well as the inhibitory role of XIAP on downstream executioner caspases. In MCF-7 cells, CASP3 is depicted as a non-functional node with disrupted upstream connections, consistent with the 47 bp exon 3 deletion that abrogates functional Caspase-3 protein expression in this cell line. These network-level findings are in full agreement with the qRT-PCR and immunofluorescence results, reinforcing the observation that Aloin preferentially activates the apoptotic machinery in cancer cells, while PNT-A1 normal epithelial cells showed no statistically significant response under identical in vitro conditions.

### 2.8. Quantitative Immunofluorescence Analysis (CTCF)

To provide quantitative support for the immunofluorescence observations, Corrected Total Cell Fluorescence (CTCF) was calculated from immunofluorescence images using ImageJ software for all four apoptotic markers across PNT-A1, MCF-7, and PC-3 cell lines (Figure 5B, Figure 6B, Figure 7B and Figure 8B). In PNT-A1 normal prostate epithelial cells, CTCF values for BAX, CASP3, CASP8, and CASP9 showed no statistically significant difference between control and Aloin-treated groups (*p* > 0.05, ns), confirming the absence of apoptotic protein upregulation in normal cells. In contrast, Aloin treatment induced highly significant increases in CTCF values in cancer cell lines. In PC-3 cells, BAX (≈38 CTCF units), CASP3 (≈22 CTCF units), CASP8 (≈34 CTCF units), and CASP9 (≈33 CTCF units) were all significantly elevated compared to controls (*p* < 0.0001, ****). In MCF-7 cells, BAX (≈20 CTCF units), CASP8 (≈6 CTCF units), and CASP9 (≈13 CTCF units) were significantly increased following Aloin treatment (*p* < 0.0001, ****), while CASP3 was not assessed in MCF-7 due to the known functional deletion. These quantitative findings are fully concordant with the qRT-PCR gene expression data and confirm that Aloin preferentially induces apoptotic protein accumulation in cancer cells while PNT-A1 normal epithelial cells showed no statistically significant response under identical in vitro conditions.

## 3. Discussion

The present study demonstrates, to the best of our knowledge for the first time, that Aloin exerts differential cytotoxic and pro-apoptotic effects in prostate cancer cells (PC-3) under in vitro conditions, while simultaneously confirming its antiproliferative activity in breast cancer cells (MCF-7). Critically, normal prostate epithelial cells (PNT-A1) remained largely unaffected across all experimental endpoints, including cell viability, morphology, apoptotic gene expression, and protein-level immunofluorescence signals. These in vitro findings collectively suggest that Aloin exhibits a preferential activity profile toward epithelial-derived cancer cells relative to normal epithelial cells, warranting further mechanistic and translational investigation.

### 3.1. Differential Cytotoxicity and IC50 Considerations

The CCK-8 viability assay revealed dose-dependent cytotoxicity in both MCF-7 and PC-3 cells, with IC50 values falling within or near the tested concentration range (1000–1500 µg/mL), while PNT-A1 cells retained viability exceeding 50% across all conditions. Time-dependent cytotoxicity was clearly evident in MCF-7 cells, whereas PC-3 cells exhibited an atypical pattern of greater sensitivity at 24 h relative to 48 h. These results are consistent with prior reports of Aloin-mediated growth inhibition in MCF-7 cells by Esmat et al. (2006), who similarly observed dose-dependent antiproliferative effects at comparable concentration ranges [20]. The IC50 values obtained here are higher than those reported for classical chemotherapeutic agents, a pattern consistently observed with anthraquinone glycosides in in vitro monolayer culture models, where bioavailability is not constrained by pharmacokinetic barriers. It should be noted that the concentration range employed in the present study (1000–1500 µg/mL) was determined following preliminary dose-finding experiments at lower concentrations, in which no significant cytotoxic or apoptotic response was observed in any of the tested cell lines. The concentration range was therefore escalated to identify biologically active conditions under in vitro monolayer culture, and is consistent with ranges reported in analogous in vitro studies employing anthraquinone glycosides [20,22,26]. While these concentrations exceed typical plasma levels achievable through conventional administration routes, in vitro monolayer models inherently require higher concentrations due to the absence of pharmacokinetic processing, protein binding, and tissue distribution effects that characterize in vivo exposure. The present study is strictly an in vitro mechanistic investigation and does not make direct translational claims regarding clinical achievability of these concentrations. Regarding solubility, Aloin was prepared as a 500 mg/mL stock solution in DMSO, with final working concentrations achieved by dilution in cell culture medium to a final DMSO concentration not exceeding 0.1% *v*/*v*—a concentration well established to have no effect on cell viability or gene expression. Regarding stability, while formal HPLC-based stability quantification was not performed in the present study, the known susceptibility of C-glycoside anthraquinones to gradual degradation at physiological temperature was proactively addressed through a medium renewal protocol applied every 24 h throughout the treatment period. This approach ensured consistent replenishment of bioactive compound and is consistent with standard practice in anthraquinone in vitro research [20,22,26]. Formal stability characterization using HPLC or UV-Vis spectrophotometry is acknowledged as a methodological enhancement recommended for future investigations. It is noteworthy that Aloin, as a C-glycoside anthraquinone, may undergo metabolic conversion in vivo to its aglycone form (aloe-emodin), which has been reported to possess greater cytotoxic potency, potentially improving effective concentrations in biological systems relative to the parent compound [12]. Furthermore, recent advances in tumor-targeted nano-delivery systems—including lipid nanoparticles, exosome-based carriers, and polymeric micelles—offer a scientifically credible perspective through which locally elevated drug concentrations could be achieved within the tumor microenvironment in future in vivo studies, potentially bridging the gap between the in vitro concentrations employed here and therapeutically achievable levels.

The Selectivity Index (SI > 1.30 for MCF-7 at 48 h) represents a minimum estimate of differential cytotoxicity, given that PNT-A1 IC50 was not reached within the tested concentration range. It is important to clarify that SI calculation in this study was not intended as a drug development selectivity assessment—rather, its sole purpose was to quantitatively document the observed differential response between cancer and normal cells under identical in vitro conditions, consistent with the exploratory and mechanistic nature of this investigation. While this value does not reach the conventional threshold of ≥3.0 cited in drug screening protocols, future studies employing nanoencapsulated Aloin formulations or combination strategies may substantially improve apparent selectivity by enhancing intracellular bioavailability in cancer cells. Notably, PC-3 cells displayed greater cytotoxic sensitivity at 24 h than at 48 h, a pattern distinct from that observed in MCF-7 cells. This may reflect the aggressive androgen-independent phenotype of PC-3 cells, which are known to upregulate adaptive survival mechanisms—including drug efflux pathways—upon prolonged cytotoxic stress. Furthermore, despite medium refreshment every 24 h, the chemical instability of Aloin as a C-glycoside anthraquinone at physiological temperature may result in progressive reduction in bioactive concentrations within each 24 h window, potentially attenuating the cumulative cytotoxic effect at 48 h. Importantly, despite this 48 h viability observation, the robust upregulation of CASP8 (11.31×), CASP9 (9.27×), and CASP3 (3.03×) at the transcriptional level confirms that Aloin-induced apoptotic signaling was firmly established in PC-3 cells, suggesting that the attenuated CCK-8 signal at 48 h reflects partial metabolic recovery in a subpopulation of surviving cells rather than true pharmacological resistance.

### 3.2. Morphological Hallmarks of Apoptosis

DIC microscopy provided orthogonal morphological evidence supporting the biochemical findings. Aloin-treated MCF-7 and PC-3 cells displayed classical apoptotic morphology including cellular rounding, volume reduction, loss of substrate adherence, and apoptotic body formation—changes consistent with phosphatidylserine externalization and cytoskeletal collapse that occur during caspase-mediated cell dismantling. In contrast, PNT-A1 cells retained intact monolayer structure and normal epithelial morphology following the same treatment, corroborating the differential response observed in the viability assays. Similar morphological discrimination between cancerous and normal cells has been reported for structurally related anthraquinone compounds [12], suggesting a class effect among anthraquinone-based phytochemicals. High-magnification immunofluorescence imaging of Aloin-treated PC-3 cells (Figure 9) further corroborated these observations, revealing intense cytoplasmic Caspase-3 and Caspase-9 signal accumulation alongside nuclear DNA fragmentation evidenced by irregular and condensed 7-AAD staining. These morphological features are consistent with caspase-mediated apoptotic cell death and are clearly distinct from necrosis, which is characterized by cellular swelling and membrane rupture—neither of which was observed in the present images.

### 3.3. Activation of the Intrinsic Apoptotic Pathway

The significant upregulation of BAX and CASP9 at both mRNA and protein levels in Aloin-treated MCF-7 and PC-3 cells strongly implicates the mitochondrial (intrinsic) apoptotic pathway as a primary mechanism. BAX, a pro-apoptotic member of the BCL-2 family, promotes mitochondrial outer membrane permeabilization (MOMP), leading to cytochrome c release and subsequent apoptosome formation with APAF1, which in turn activates procaspase-9. The concordant upregulation of both BAX and CASP9 across both cancer cell lines suggests that Aloin disrupts the balance between pro- and anti-apoptotic BCL-2 family members in favor of MOMP induction. These findings align with earlier reports in non-small-cell lung cancer, where Aloin was shown to promote apoptosis through ROS-mediated mechanisms [21,26], and with the work of Gao et al. (2022), who implicated PI3K/AKT axis modulation in Aloin-induced gastric cancer apoptosis [28]—a pathway that directly impinges on BCL-2 family member phosphorylation and BAX translocation.

### 3.4. Activation of the Extrinsic Apoptotic Pathway

Beyond the intrinsic route, the present study provides evidence for concurrent extrinsic pathway activation, as demonstrated by the significant upregulation of CASP8 at both mRNA and protein levels in both cancer cell lines. Caspase-8 is the apical initiator caspase of the death receptor pathway, classically activated downstream of FADD recruitment to the death-inducing signaling complex (DISC) upon ligand engagement of receptors such as FAS, TRAIL-R1, or TRAIL-R2. The simultaneous activation of both initiator caspases (Caspase-8 and Caspase-9) observed here suggests that Aloin may engage multiple apoptotic entry points or, alternatively, that crosstalk between pathways—mediated through BID cleavage by Caspase-8 to truncated BID (tBID), which then amplifies mitochondrial apoptosis—may be operative. Future mechanistic studies incorporating BID knockdown or FLIP overexpression models could help delineate the relative contributions of primary extrinsic signaling versus amplification loops in Aloin-treated cells.

### 3.5. Effector Caspase Activation and the MCF-7 CASP3 Limitation

In PC-3 cells, all four apoptotic markers—BAX, CASP9, CASP8, and CASP3—were significantly upregulated at the mRNA level following Aloin treatment, confirming downstream execution-phase caspase activation and the completion of a functional apoptotic cascade. CASP3 mRNA analysis was appropriately excluded from MCF-7 cells owing to the well-characterized 47 bp deletion in exon 3 of the CASP3 gene, which abolishes functional Caspase-3 protein expression in this cell line. This genetic deficiency in MCF-7 cells does not, however, preclude apoptosis per se, as alternative executioner caspases such as Caspase-7 may compensate, and caspase-independent cell death mechanisms—including AIF nuclear translocation or cathepsin release—may also contribute. The significant upregulation of CASP9 and CASP8 in MCF-7 cells indicates that Aloin initiates apoptotic signaling upstream of the executioner level.

### 3.6. Cancer Cell Selectivity: Mechanistic Hypotheses

The observation that PNT-A1 normal cells remained unaffected at both the transcriptional and protein levels while cancer cells showed robust apoptotic induction warrants mechanistic interpretation. Several non-mutually exclusive explanations may account for this selectivity. First, cancer cells frequently exhibit higher baseline reactive oxygen species (ROS) levels and greater mitochondrial membrane potential instability compared to normal cells; Aloin’s capacity to further elevate ROS—as demonstrated in lung cancer models [21,26]—may preferentially push transformed cells past a cell death threshold while normal cells maintain redox homeostasis through intact antioxidant defense systems. Second, the differential expression of death receptors and their downstream effectors in cancer versus normal epithelium may render cancer cells intrinsically more susceptible to extrinsic apoptotic stimuli. Third, the dysregulation of BCL-2/BCL-XL expression commonly observed in MCF-7 and PC-3 cells may lower the threshold for Aloin-induced MOMP compared to PNT-A1 cells, in which antiapoptotic safeguards remain functionally intact.

### 3.7. Comparison with Existing Literature

Aloin’s anticancer activities have previously been characterized across a variety of malignancies. In colorectal cancer, Pan et al. (2013) reported inhibition of VEGF-driven angiogenesis and STAT3 pathway suppression [24], while gastric cancer studies identified NOX2-ROS and Akt/mTOR signaling as downstream targets [25,28]. In non-small-cell lung cancer, Aloin induced dose-dependent apoptosis in A549 and H1299 cells [26], and in melanoma it modulated HMGB1 expression [27]. Esmat et al. (2006) previously reported cytotoxicity of Aloin in MCF-7 cells via topoisomerase IIα inhibition [20], and Ahmed et al. (2023) demonstrated autophagy induction in the same cell line in comparison with doxorubicin [30]. The present study extends this body of evidence by demonstrating, for the first time, prostate cancer cell sensitivity to Aloin and by providing a mechanistic dissection of apoptotic pathway activation, simultaneously characterizing both intrinsic and extrinsic routes in these cell types. Furthermore, the inclusion of a normal cell comparator and the generation of selectivity index data represent methodological advances relative to several prior studies. Collectively, the STRING-based PPI network analysis (Figure 10) visually consolidates these findings by mapping the four experimentally validated genes onto the established apoptosis network, confirming their central roles within both the intrinsic and extrinsic pathways and highlighting key regulatory nodes—including BID-mediated pathway crosstalk and XIAP-dependent executioner caspase inhibition—that align with the mechanistic framework discussed herein. Importantly, quantitative immunofluorescence analysis using ImageJ-based CTCF measurements provided objective, numerical confirmation of the protein-level findings, demonstrating highly significant fluorescence intensity increases in Aloin-treated PC-3 and MCF-7 cells (*p* < 0.0001) while PNT-A1 cells remained unaffected. This addresses the inherent semi-quantitative limitation of conventional immunofluorescence and strengthens the validity of the protein expression conclusions. Furthermore, pathway enrichment analysis across three independent databases (KEGG 2026, Reactome 2024, and WikiPathways 2024) consistently demonstrated that the four selected genes are statistically central to conserved apoptotic signaling networks, with adjusted *p*-values reaching as low as 10^−11^ (Figure 3). The concurrent enrichment of both intrinsic and extrinsic apoptotic pathway terms corroborates the experimental findings, while significant enrichment of prostate cancer- and breast cancer-specific pathways—including miRNA Regulation of p53 Pathway in Prostate Cancer and Androgen Receptor Network in Prostate Cancer—provides independent bioinformatic validation for the selection of PC-3 and MCF-7 cell lines and the choice of these four apoptotic markers in this study.

### 3.8. Limitations and Future Directions

The present study was designed as a foundational, hypothesis-driven investigation to establish, for the first time, the cytotoxic and apoptotic profile of Aloin in prostate and breast cancer cell lines using a well-validated multimodal in vitro framework. Within this defined scope, several limitations merit consideration, each of which identifies specific directions for subsequent independent investigations rather than undermining the validity of the current findings. First, the absence of a tissue-matched normal breast epithelial cell line (e.g., MCF-10A) is acknowledged. However, the principal active metabolite of Aloin, aloe-emodin, has been shown to spare non-tumorigenic MCF-10A cells at anticancer concentrations [32,33], and both MCF-7 and PNT-A1 share epithelial origin, supporting the biological validity of the selectivity comparison made here. Incorporation of MCF-10A in future work would further substantiate this observation. Second, while the current study employed immunofluorescence with quantitative CTCF analysis to demonstrate protein-level expression changes, Western blotting for cleaved caspase forms and flow cytometry-based Annexin V/PI assays were not included in the original project scope. This reflects a deliberate design decision consistent with the foundational and exploratory nature of this investigation, rather than an unplanned omission. The present project was designed with defined endpoints—namely, the first characterization of Aloin’s cytotoxic and apoptotic profile in prostate cancer cells using a multimodal approach—and the convergent evidence from qRT-PCR, spatial immunofluorescence, quantitative CTCF measurements, high-magnification morphological imaging (Figure 9), and DIC microscopy collectively constitutes a multi-layered apoptotic profile that fully addresses the research hypotheses set forth in this study. Formal confirmation of cleaved caspase species via Western blotting, Annexin V/PI-based apoptotic quantification by flow cytometry, and pharmacological blockade experiments using specific caspase inhibitors (e.g., Z-VAD-FMK, Z-IETD-FMK) are identified as the primary endpoints of a planned follow-up investigation, in which these complementary approaches will be employed as dedicated mechanistic tools. We wish to emphasize that the findings of the present study do not merely represent an end point—they constitute the scientific foundation upon which these more in-depth mechanistic investigations will be built. The robust multi-endpoint evidence generated here establishes the biological rationale and provides the necessary preliminary data to justify and design future studies incorporating Western blotting, flow cytometry, mitochondrial membrane potential assays, and in vivo validation. In this sense, the present study and its planned follow-up are viewed as complementary phases of a broader research program aimed at comprehensively elucidating the anticancer mechanism of Aloin in epithelial-derived malignancies. Third, assessment of mitochondrial outer membrane permeabilization (MOMP) and membrane potential dynamics—for example via JC-1 or TMRE staining—would provide direct evidence of intrinsic pathway engagement at the organellar level and is likewise proposed as a component of future work. Finally, as an in vitro study, the present findings do not address the pharmacokinetic behavior of Aloin in vivo, including its oral bioavailability, metabolic conversion to aloe-emodin, plasma protein binding, and achievable intratumoral concentrations. In vivo validation using appropriate xenograft or orthotopic tumor models, together with nano-delivery formulation strategies to optimize bioavailability, constitutes the natural extension of this work and is identified as the primary objective of a planned follow-up investigation. Additionally, molecular docking analysis between Aloin and the apoptotic target proteins identified in this study (BAX, CASP3, CASP8, CASP9)—including binding pose analysis, energy minimization, and molecular dynamics simulation—is identified as a priority objective of a planned follow-up computational investigation, which would provide mechanistic insight at the molecular interaction level to complement the network pharmacology framework established in the present study. Collectively, the present study establishes the cellular and molecular foundation upon which these mechanistic and translational questions will be systematically addressed.

## 4. Material and Methods

### 4.1. Preparation of Aloin Solutions

The preparation of aloin (“Cayman Chemical, Ann Arbor, MI, USA, Cat. No. 22435”) solutions involved making a 500 mg/mL stock solution using DMSO (“Calbiochem, San Diego, CA, USA, Cat. No. 317275-500”) as the solvent. The stock solution was diluted in cell culture media to final working concentrations of 1000 µg/mL, 1250 µg/mL, and 1500 µg/mL. To ensure sterility, the resulting solutions were filtered through 0.22 µm sterile filters (“Sartorius AG, Göttingen, Germany”) and transferred to sterile Eppendorf tubes. The final DMSO concentration did not exceed 0.1% (*v*/*v*) in any experimental or control group throughout all assays, a concentration well below the threshold known to affect cell viability or gene expression.

### 4.2. Cell Culture

Human prostate cancer cell line PC-3 (ATCC, CRL-1435), human normal prostate epithelial cell line PNT-A1 (ECACC, 95012613), and human breast cancer cell line MCF-7 (ATCC, HTB-22) were cultured in DMEM high glucose medium (“Sigma, St. Louis, MO, USA, Cat. No. D6429-500ML”) supplemented with 10% heat-inactivated fetal bovine serum (FBS) (“Biowest, Nuaillé, France, Cat. No. S181H-500”), 1% L-glutamine (“Gibco, Thermo Fisher Scientific, Waltham, MA, USA, Cat. No. 25030081”), and 1% penicillin-streptomycin (10,000 U/mL–10,000 µg/mL) (“Gibco, Thermo Fisher Scientific, Waltham, MA, USA, Cat. No. 10378016”). All cell lines were maintained at 37 °C in a humidified atmosphere containing 5% CO_2_.

### 4.3. Morphological Analysis by Differential Interference Contrast (DIC) Microscopy

Differential Interference Contrast (DIC) microscopy was employed to assess Aloin-induced morphological alterations in PC-3, PNT-A1, and MCF-7 cells. Cells were seeded onto sterile glass coverslips in 6-well plates and grown to approximately 70–80% confluence prior to treatment. Aloin was administered at final concentrations of 1000, 1250, and 1500 µg/mL, and cultures were incubated for 24 h and 48 h under standard conditions (37 °C, 5% CO_2_). At each time point, cells were gently rinsed with pre-warmed PBS, and live-cell imaging was performed immediately using a DIC-equipped inverted microscope (Zeiss Axio Observer (Carl Zeiss AG, Oberkochen, Germany)) without fixation to prevent morphological artifacts. Images were acquired from multiple random fields using consistent illumination and exposure settings to allow reliable comparison across treatment groups. Morphological evaluation focused on treatment-associated changes including alterations in cell size, cytoplasmic density, adherence, membrane structure, and nuclear–cytoplasmic contrast.

### 4.4. Cytotoxicity Assay: CCK-8

MCF-7, PC-3, and PNT-A1 cell lines were seeded at 5500 cells per well in 96-well plates and incubated overnight at 37 °C in a 5% CO_2_ incubator. The medium was then removed and replaced with Aloin-containing media at the designated concentrations (see Preparation of Aloin Solutions). For the control group, medium containing the equivalent volume of DMSO vehicle (<0.1% *v*/*v*; “Calbiochem, 317275-500”) was used. To prevent degradation of Aloin, treatment medium was refreshed every 24 h. Cell viability was assessed using the CCK-8 kit (WST-8 reagent; Dojindo Molecular Technologies, Kumamoto, Japan). The tetrazolium salt WST-8 contained in CCK-8 is reduced by cellular dehydrogenases to a formazan dye proportional to the number of metabolically active cells. Absorbance was measured at 450 nm using a Tecan Infinite 200 PRO microplate reader (Tecan Group Ltd., Männedorf, Switzerland).

### 4.5. IC50 and Selectivity Index (SI) Analysis

IC50 values were estimated from dose–response cell viability data obtained after 24 h and 48 h exposure in PNT-A1, MCF-7, and PC-3 cells across the tested concentration range (1000, 1250, and 1500 µg/mL) using GraphPad Prism 9 software (GraphPad Software, San Diego, CA, USA). When cell viability did not decrease to 50% within the tested concentration range, the IC50 was reported as >1.5 mg/mL. The Selectivity Index (SI) was calculated as the ratio of the IC50 value in PNT-A1 normal cells to that in cancer cells (SI = IC50 [normal]/IC50 [cancer]); values were expressed as minimum estimates when the IC50 in PNT-A1 cells was not reached within the tested range, and interpreted as lower-bound estimates of selectivity.

### 4.6. Pathway Enrichment Analysis

Pathway enrichment analysis was performed using the Enrichr web-based bioinformatics platform (https://maayanlab.cloud/Enrichr, accessed on 2 June 2026) [34,35]. The four experimentally validated target genes (BAX, CASP3, CASP8, and CASP9) were submitted as input to three independent pathway databases: KEGG 2026, Reactome 2024, and WikiPathways 2024 Human. Statistical significance was determined using Fisher’s exact test with Benjamini–Hochberg correction for multiple comparisons. Only pathways with adjusted *p*-value < 0.05 were considered statistically significant. Enrichment results were exported as tab-delimited text files from the Enrichr interface. Bubble plot visualization was generated using Python (version 3.11, Python Software Foundation, https://www.python.org, accessed on 2 June 2026)with the Matplotlib (version 3.7.1) library [36]. Bubble size represents the number of query genes matched to each pathway (gene overlap), and color intensity reflects the statistical significance expressed as −log_10_ (adjusted *p*-value).

### 4.7. Gene Expression Analysis by qRT-PCR

Total RNA was extracted from MCF-7, PC-3, and PNT-A1 cells following 48 h treatment with 1250 µg/mL Aloin or equivalent DMSO vehicle control (<0.1% *v*/*v*) using TRIzol Reagent (Invitrogen) according to the manufacturer’s instructions. Briefly, cells were lysed directly in TRIzol, followed by phase separation with chloroform, RNA precipitation with isopropanol, and washing with 75% ethanol. The RNA pellet was dissolved in RNase-free water. RNA concentration and purity were assessed spectrophotometrically (NanoDrop, Thermo Fisher Scientific, Waltham, MA, USA) samples with A_260_/A_280_ ratios between 1.8 and 2.0 were considered acceptable. Complementary DNA (cDNA) was synthesized from 1 µg of total RNA using the iScript cDNA Synthesis Kit (Bio-Rad, catalog no. 1708891) according to the manufacturer’s protocol. Quantitative real-time PCR (qRT-PCR) was performed using SsoFast EvaGreen Supermix (Bio-Rad, catalog no. 172-5201) on a CFX96 Real-Time PCR Detection System (Bio-Rad Laboratories, Hercules, CA, USA), following the manufacturer’s recommended cycling conditions. The expression levels of CASP3, CASP8, CASP9, and BAX were analyzed, with GAPDH as the reference gene for normalization. Amplification efficiency for all primer pairs was validated and confirmed to be within the accepted range of 90–110%, ensuring reliable quantification. Primer sequences used in this study are listed in Table 2. Relative mRNA expression was calculated using the 2^−ΔΔCt^ method, where ΔCt = Ct(target gene) − Ct(GAPDH), and ΔΔCt = ΔCt(Aloin-treated) − ΔCt(vehicle control). Relative expression values (2^−ΔΔCt^) were subsequently log_2_-transformed to yield log_2_ fold change (log_2_FC) values for heatmap visualization, where log_2_FC = log_2_(2^−ΔΔCt^) = −ΔΔCt. Fold change values (×) were calculated directly as 2^−ΔΔCt^. All experiments were performed with three independent biological replicates (n = 3), each analyzed in technical triplicate, and results are expressed as mean ± SD. Due to the well-characterized 47-base pair deletion in exon 3 of the CASP3 gene in MCF-7 cells, which abrogates functional Caspase-3 protein expression, CASP3 mRNA expression analysis was performed exclusively in PC-3 and PNT-A1 cell lines.

### 4.8. Immunofluorescence Analysis: BAX, Caspase-3, Caspase-8, Caspase-9

Cell lines were cultured on coverslips in 24-well plates. For mechanistic analyses, the concentration of 1250 µg/mL at 48 h was selected based on CCK-8 dose–response data as the most biologically informative treatment condition, representing the concentration that produced significant and reproducible cytotoxic effects in cancer cells while maintaining the differential response relative to PNT-A1 normal cells. This selection is consistent with established practice in phytochemical apoptosis studies. Cells treated with 1250 µg/mL Aloin-containing medium or equivalent DMSO vehicle control (<0.1% *v*/*v*) were fixed with 3.5% paraformaldehyde (“Sigma, 158127”) after 48 h. Fixed coverslips were washed with PBS (“Gibco, 14190-094”) and preserved in PBS-azide (“Chemcruz, SC-296028”) at +4 °C. Cells were then incubated with primary antibodies against Caspase-3 (“Abcam, ab13847, rabbit”), Caspase-9 (“Abcam, ab202068, rabbit”), Caspase-8 (“Bioss, bs-0052r, rabbit”), and BAX (“Abcam, ab32503, rabbit”) for 24 h at +4 °C. Following PBS washes to remove unbound antibody, FITC-conjugated anti-rabbit secondary antibodies (“Sigma, F9887”) were applied and incubated for 1 h at 37 °C. After further PBS washing, cells were stained with 7-aminoactinomycin D (7-AAD) (“Invitrogen, A1310”) for nuclear counterstaining and incubated for 15 min at room temperature in the dark. 7-AAD, a cell-impermeant DNA intercalating agent with far-red emission (λ_em_ ≈ 647 nm), was selected as a nuclear counterstain spectrally compatible with FITC-labeled secondary antibodies, enabling unambiguous co-localization of nuclear and cytoplasmic fluorescence signals in multicolor immunofluorescence imaging. Coverslips were washed with PBS, mounted with ProLong Gold antifade mounting medium (Thermo Fisher Scientific, P36930), sealed, and stored at −20 °C until imaging. All immunofluorescence images were acquired using a fluorescence microscope at 40× magnification.

To provide quantitative fluorescence intensity data, Corrected Total Cell Fluorescence (CTCF) was calculated from immunofluorescence images using ImageJ software (version 1.54, National Institutes of Health, USA) [37]. For each experimental group, a minimum of ten cells were individually selected as regions of interest (ROIs). Background fluorescence noise was eliminated using a minimum of three background measurements per image taken from cell-free areas. CTCF was calculated using the formula: CTCF = Integrated Density − (Area of selected cell × Mean fluorescence of background readings). Statistical comparisons between control and Aloin-treated groups were performed using an unpaired two-tailed Student’s *t*-test. Results are presented as mean ± SD, and significance was denoted as follows: ns = not significant; **** *p* < 0.0001.

### 4.9. Heatmap Visualization of Gene Expression Data

To provide an integrated visual summary of the qRT-PCR results across all cell lines and genes, a heatmap was generated using Python 3 (Python Software Foundation) with the matplotlib and seaborn libraries. Relative mRNA expression values (2^−ΔΔCt^) for each gene (CASP8, CASP9, BAX, CASP3) and cell line (PNT-A1, PC-3, MCF-7) were used to calculate fold change relative to the vehicle control group (Aloin/Control). Fold changes were subsequently log_2_-transformed to generate log_2_ fold change (log_2_FC) values, which were used to encode color intensity using a diverging Red–Blue color scale (blue: no change; red: upregulation). Fold change (×) and log_2_FC values were annotated within each heatmap cell, and statistical significance labels derived from one-way ANOVA with Tukey’s post hoc test were overlaid accordingly. CASP3 data were excluded from MCF-7 cells due to the known 47 bp exon 3 deletion, and the corresponding cell was marked as N/A.

### 4.10. Protein–Protein Interaction (PPI) Network Analysis

To contextualize the experimentally validated apoptotic genes within the established human apoptosis signaling network, a protein–protein interaction (PPI) network analysis was performed using the STRING database (version 12.0, Homo sapiens). The four core genes quantified by qRT-PCR in this study (CASP8, CASP9, BAX, and CASP3) were used as seed proteins, and first-degree interactors were retrieved applying a combined confidence score threshold of ≥0.700. The resulting network was visualized using Python (networkx and matplotlib libraries), with core gene nodes colored according to fold change values relative to the vehicle control group. In MCF-7 cells, CASP3 was represented as a non-functional node with disrupted downstream connections, consistent with the known 47 bp deletion in exon 3 of the CASP3 gene in this cell line.

### 4.11. Statistical Analysis

Experiments were conducted according to completely randomized design (CRD) principles. GraphPad Prism 9 software was used for all statistical analyses. The normality of data distribution was assessed using the Shapiro–Wilk and Kolmogorov–Smirnov tests. Data meeting normality criteria were evaluated by one-way ANOVA followed by Tukey’s post hoc test for multiple comparisons. Results with *p* values below 0.05 (*p* < 0.05) were considered statistically significant. All experiments were performed in at least three independent replicates, and data are presented as mean ± SD.

## 5. Conclusions

The present study provides the first in vitro evidence of Aloin’s cytotoxic and pro-apoptotic activity in prostate cancer cells (PC-3), and further corroborates its antiproliferative effects in breast cancer (MCF-7), while demonstrating that normal prostate epithelial cells (PNT-A1) are largely spared under the same in vitro conditions. Collectively, our findings demonstrate that Aloin induces dose-dependent cytotoxicity preferentially in MCF-7 and PC-3 cancer cell lines, triggers hallmark apoptotic morphological changes, and activates both intrinsic and extrinsic apoptotic pathways, as evidenced by significant upregulation of BAX, CASP9, and CASP8 at both mRNA and protein levels across both cancer cell lines, and CASP3 at mRNA and protein levels in PC-3 cells. The concordance between qRT-PCR, quantitative CTCF immunofluorescence, DIC morphological analysis, and high-magnification immunofluorescence imaging (Figure 9) strengthens confidence in these conclusions, with the latter providing morphological evidence consistent with caspase-mediated apoptotic cell death rather than necrosis. Pathway enrichment analysis across three independent databases (KEGG 2026, Reactome 2024, and WikiPathways 2024) independently confirmed these genes as statistically central nodes in conserved apoptotic signaling networks, providing bioinformatic validation for the experimental findings. These in vitro findings establish a mechanistic foundation for Aloin as a phytochemical of interest in epithelial-derived malignancies. All findings are strictly confined to in vitro conditions and do not constitute translational claims. Future studies should focus on in vivo validation in appropriate tumor models, mechanistic dissection of upstream signaling events including caspase activation and mitochondrial membrane dynamics, and assessment of nano-delivery formulations to optimize bioavailability and advance the translational potential of these findings.

## Figures and Tables

**Table 1:** Estimated IC50 and Selectivity Index (SI) values of Aloin in PNT-A1, MCF-7, and PC-3 cells.

Cell Line	Exposure Time	IC_50_ (mg/mL)	SI
PNT-A1	24 h	>1.5	—
PNT-A1	48 h	>1.5	—
MCF-7	24 h	>1.5	ND
MCF-7	48 h	1.15 ^†^	>1.30
PC-3	24 h	1.49 ^†^	>1.0
PC-3	48 h	>1.5	ND

^†^ Estimated within the tested concentration range. ND: Not determined (IC_50_ not reached within the tested concentration range; applies to MCF-7 at 24 h and PC-3 at 48 h).

**Table 2:** Primer sequences used for qRT-PCR analysis.

Gene	Direction	Sequence (5′ → 3′)	Role
CASP9	Forward	CCTGTGTCGGTCGAGAAGAT	Target gene
	Reverse	TGGGTGTGGGCAAACTAGAT	
CASP8	Forward	AGAGTCTGTGCCCAAATCAACA	Target gene
	Reverse	AAGGCTGCTGCTTCTCTCTTTG	
CASP3	Forward	ATGGAAGCGAATCAATGGA	Target gene
	Reverse	TGTACCAGACCGAGATGTC	
BAX	Forward	CTTTTGCTTCAGGGTTTCATCC	Target gene
	Reverse	TGAAGTTGCCGTCAGAAAACAT	
GAPDH	Forward	5′-TGCACCACCAACTGCTTAG-3′	Reference gene
	Reverse	5′-GATGCAGGGATGATGTTC-3′	

## Data Availability

The original contributions presented in this study are included in the article. Further inquiries can be directed to the corresponding author.
